# Primary diffuse large B-cell tumor of the uterus: a case report

**DOI:** 10.3389/fmed.2023.1234861

**Published:** 2023-07-13

**Authors:** Zhiming Zhang, Qing Yang

**Affiliations:** Department of Obstetrics and Gynecology, Shengjing Hospital of China Medical University, Shenyang, China

**Keywords:** uterus, diffuse large B-cell tumor, extra-nodal lymphoma, diagnosis, treatment

## Abstract

Primary diffuse large B-cell tumor of the uterus is a rare clinical condition with a similar clinical presentation to gynecologic tumors and is easily misdiagnosed. We report a case of a postmenopausal woman who presented with pelvic mass. Her ultrasound and MRI examinations suggested diffuse enlargement of the uterus, leading us to consider the diagnosis of lymphoma. The histological examination of the uterine mass confirmed the diagnosis of diffuse large B-cell lymphoma. The patient received 6 cycles of chemotherapy with R-CHOP (rituximab, cyclophosphamide, doxorubicin, vincristine and prednisone) and 2 cycles of consolidation therapy with R (rituximab). After treatment, the patient's uterus was significantly smaller than before and there was no sign of a recurrence.

## Introduction

Lymphoma is a malignant neoplasm originating from lymphocytes in lymph nodes or extra-nodal lymphoid tissue and can be classified into Hodgkin's lymphoma and non-Hodgkin's lymphoma (about 90% of all lymphomas) based on histopathological changes (1). Approximately 30% of non-Hodgkin's lymphomas (NHL) originate from extra-nodal sites, with the most common sites being the gastrointestinal tract, skin, bone and central nervous system (2), and only 0.2–1.1% of cases of extra-nodal lymphoma in the female genital tract (3). Lymphomas arising in the uterus account for only 16.5% of primary female genital tract lymphomas (PLFGT), and their histologic type is predominantly diffuse large B-cell lymphoma (DLBCL) (4, 5). Due to its low incidence and nonspecific clinical symptoms, uterine DLBCL is difficult to diagnose or even misdiagnose in the clinic, and there are no standard treatment options. Here we report a patient with primary uterine DLBCL and discuss case characteristics, diagnosis and treatment to improve the understanding of uterine DLBCL among gynecologists.

## Case report

**Figure 1:**
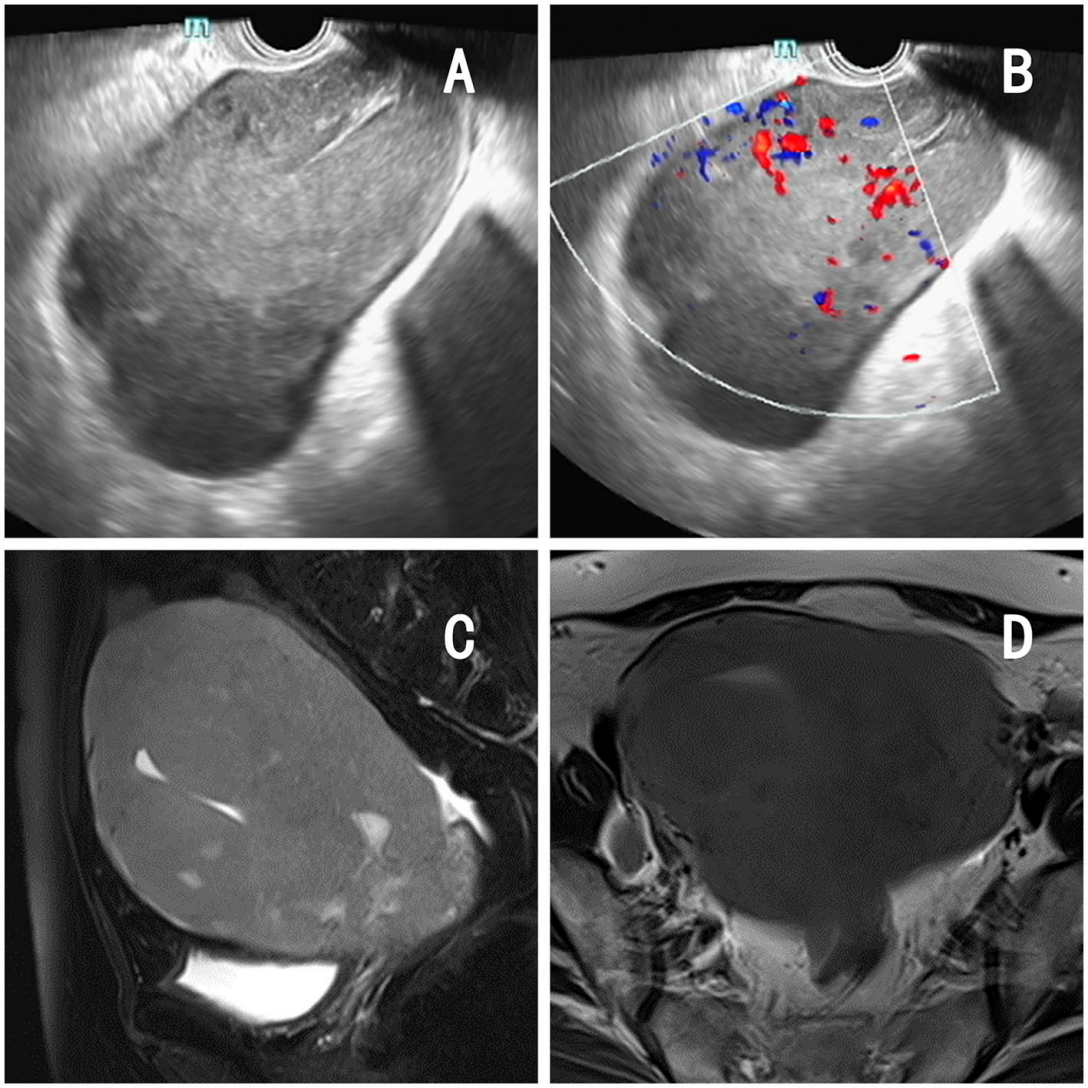
Imaging findings of the patient. **(A, B)** Pelvic ultrasound: a 13.4 × 9.4 × 8.2 cm mass in the uterine region with clear borders, with rich blood flow signal detectable on CDFI and arterial spectrum detectable. **(C, D)** Pelvic MRI: significant increase in uterine volume, thickening of the muscular wall, measuring 5.6 cm at the thickest point, accompanied by diffuse abnormal signals and enhancement changes.

**Figure 2:**
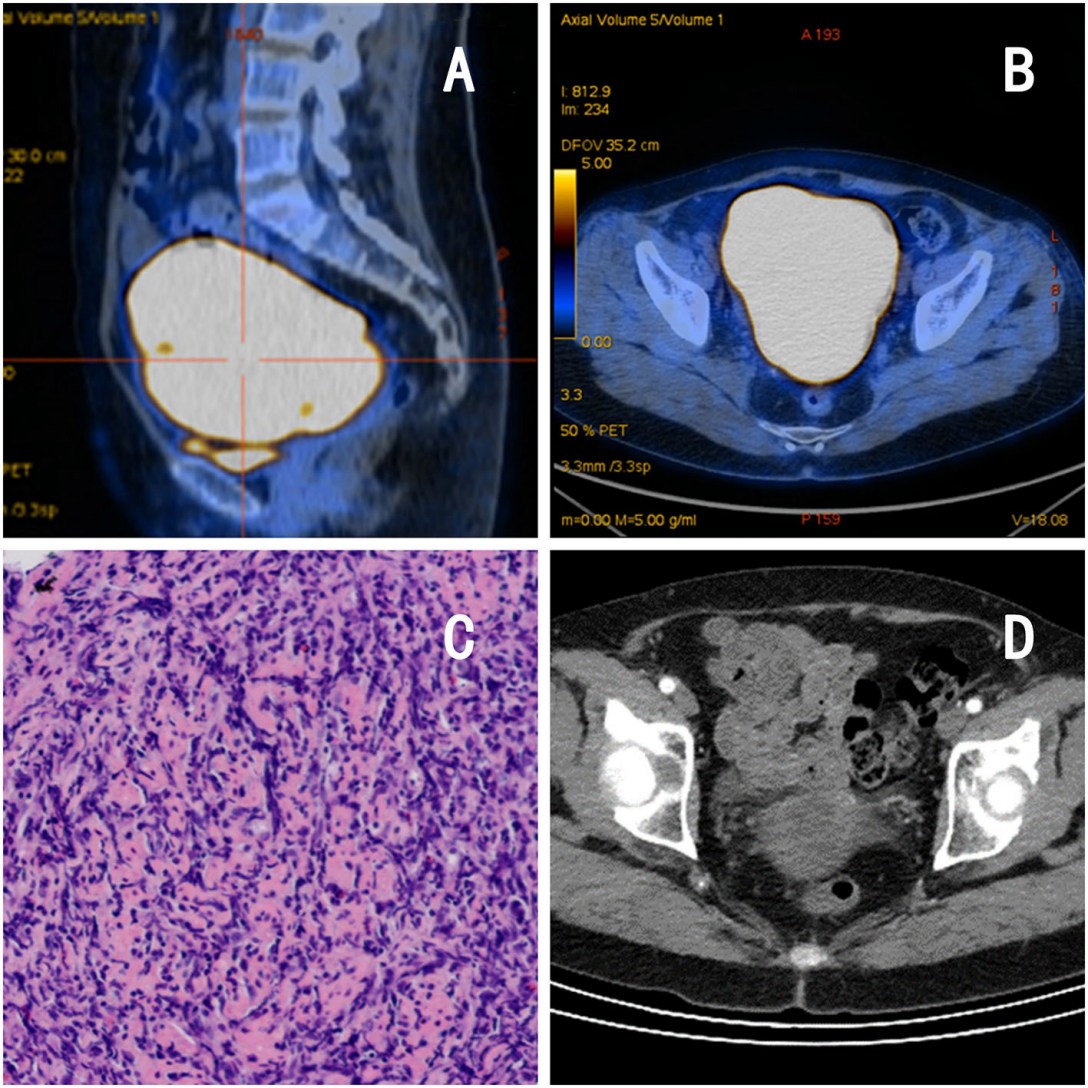
Imaging and pathological findings of the patient. **(A, B)** PET-CT: the uterus is enlarged, measuring −12.6 × 11.7 cm, with diffuse increased FDG metabolism, consistent with malignant changes. **(C)** Pathology: lymphoid cells arranged in a diffuse sheet pattern. **(D)** Abdominal CT after chemotherapy:a reduced uterine volume of −6.8 × 4.3 × 3.8 cm with less uniform density.

The patient was a 62-year-old postmenopausal woman, gravida 1, para 1, with no history of hypertension or diabetes mellitus and no history of surgery. On October 16, 2022, the patient visited our hospital for “conscious pelvic mass with hardness for 1 month, without abdominal pain.” She reported that she had not had a regular physical examination and had not been treated in other hospital prior to visiting us. On gynecological examination, the vulva and vagina were normal, a small amount of discharge was visible, the cervix was atrophic, with chronic inflammation on the surface, the uterus was obviously enlarged, hard, without pressure pain, reaching 2 fingers below the umbilicus, and no obvious abnormality was found in both adnexal areas. The pelvic ultrasound showed a 13.4 × 9.4 × 8.2 cm mass in the uterine region with clear borders, seemingly uterine morphology, hypoechoic inside, and a central endothelial line seemingly intermittent, about 1.2 cm thicker, with rich blood flow signal detectable on CDFI and arterial spectrum detectable, RI: 0.33 (Figures 1A, B). Thinprep cytology test (TCT) showed no intraepithelial lesions or malignant lesions (NILM); HPV: negative. Normal levels of alpha-fetoprotein (AFP), carcinoembryonic antigen (CEA), glycoantigen 125 (CA-125), and glycoantigen 19-9 (CA19-9). The pelvic enhancement magnetic resonance imaging (MRI) showed that the uterine volume was significantly enlarged and the muscle wall was generally thickened, with a thicker area of 5.6 cm, with a slightly longer T1 and longer T2 signal predominating, and a heterogeneous signal in the T2WI, mixed with a few small patchy high-signal shadows, and the enhancement scan showed heterogeneous mild to moderate enhancement in the myometrial wall, which seemed to be caused by multiple nodules and masses fusion, and some of them were seen as patchy non-obvious enhancement shadows (Figures 1C, D), PET-CT showed an enlarged uterus with unclear endometrium, measuring ~12.6 × 11.7 cm. There is a significant diffuse increase in FDG metabolism in the uterus with a SUVmax of 36.34 (Figures 2A, B). Metastatic lesions were observed beneath the capsule of the left outer lobe of the liver, measuring ~2.2 × 2.4 cm. There were also metastatic lesions in the 6th and 11th thoracic vertebrae. Multiple lymph node metastases were detected in the bilateral pelvic sidewalls, retroperitoneal region, and left diaphragmatic angle. Because of its specific imaging presentation, we performed an ultrasound-guided transabdominal uterine mass puncture biopsy for definitive diagnosis on the patient, and the pathology showed a non-Hodgkin diffuse large B-cell lymphoma of germinal center origin (Figure 2C). After the diagnosis of uterine lymphoma was confirmed, the patient was referred to the hematology department for further treatment. Further laboratory workup revealed the following levels: β2-MG 2.84 mg/L, LDH 421 U/L, no significant abnormalities were observed in the blood cell counts. The bone marrow cell immunophenotyping analysis revealed strong positive expression of CD20, weak positive expression of CD5, and negative expression of CD10 and Ki-67. Additionally, no abnormal monoclonal mature B lymphocytes were detected. The cerebrospinal fluid test also showed no abnormal phenotypic B lymphocytes, suggesting that the patient's lesions did not involve the nervous systems. Then she was diagnosed with Non-Hodgkin's lymphoma (Diffuse Large B-cell type), Stage IV, Group A, with an International Prognostic Index (IPI) score of 4. The treatment regimen was 6 courses of standard doses of R-CHOP (rituximab, cyclophosphamide, doxorubicin, vincristine, and prednisone) every 3 weeks. During the chemotherapy process, the patient experienced side effects such as leukopenia (reduced white blood cell count) and hair loss. After six courses of treatment, the gynecological exam revealed a significant reduction in the size of the uterus. The laboratory workup revealed the following levels: β2-MG 1.73 mg/L, LDH 258 U/L. We recommended the patient to undergo a follow-up PET-CT scan, but the patient refused (owing to financial constraints) and opted only for an enhanced abdominal CT and ultrasound examinations. The abdominal enhancement CT showed a reduced uterine volume of ~6.8 × 4.3 × 3.8 cm with less uniform density (Figure 2D). The metastatic lesion in the left lobe of the liver has also decreased in size compared to before, measuring ~1.7 × 1.8 cm. There is also a reduction in the size of the bilateral pelvic lymph nodes compared to before. The ultrasound examinations revealed no significant abnormal enlargement of lymph nodes in the bilateral axillary, supraclavicular (both above and below), neck, and inguinal regions. Three weeks after the 6th R-CHOP treatment, the patient was given 2 courses of consolidation therapy with rituximab 600 mg IV pump every 3 weeks. The patient is now in excellent general condition with no signs of recurrence.

## Discussion

Primary uterine DLBCL is an extremely rare and poorly reported disease that normally occurs in postmenopausal women (6). Its cause, like most extra-nodal lymphomas, is unknown. Patients commonly present with abnormal uterine bleeding, abdominal or pelvic discomfort (7), and in a few patients, urinary symptoms (because of bladder or ureteral compression) such as urinary frequency, urinary urgency, or hydronephrosis (8). Some asymptomatic patients are found to have an enlarged uterus during gynecologic examination, which is consistent with our patient. Typically, patients with systemic lymphoma present with systemic symptoms such as fever, night sweats and weight loss, while uterine lymphoma rarely presents with these symptoms (9).

The lack of specificity in the clinical presentation of uterine DLBCL makes it difficult to distinguish it from other tumors originating in the uterus (such as uterine fibroids and uterine sarcomas) and makes it easy to misdiagnose. Therefore, having the appropriate diagnostic tools is crucial for clear diagnosis, staging and treatment. In terms of imaging, uterine DLBCL has a certain specificity. On ultrasound, it presents as a uniformly enlarged uterus with relatively homogeneous echogenicity reduction in the myometrium, with no significant abnormalities in endometrial morphology or echogenicity, and most lesions have moderate to abundant blood flow levels on CDFI (10). On MR, it shows diffuse uterine enlargement with homogeneous signal, permeating the entire layer without disturbing the normal contour, and homogeneous low signal in the T1-weighted sequence and slightly elevated signal in the T2-weighted sequence (11). In the presence of the above imaging findings, biopsy histopathology evaluation and inventing should be used to further clarify the diagnosis. The pathological histology of uterine DLBCL showed a diffuse large cell infiltrate with relatively homogeneous morphology, and after performing immunophenotyping assays, all expressed CD20, PAX-5 and CD79a, none expressed CD3, CD43, CD45RO, and most expressed bcl-6 (84.6%), CD10 (69.2%), MUM-1 (69.2%), EMA (53.8%) (12). The patient we report presented with nothing but an enlarged uterus. Her imaging studies revealed a uniformly enlarged uterus. This relatively specific imaging finding reminds us of uterine lymphoma. Our conjecture is confirmed by the subsequent pathological findings. Therefore, when encountering such patients, a comprehensive analysis of clinical presentation, imaging, histology, and immunophenotypes must be performed to establish a correct diagnosis.

Uterine DLBCL, like other primary female genital system lymphomas, has no standard treatment options. The treatment options are the following three: (1) chemotherapy: it's the primary treatment modality, and the common chemotherapy regimens are CHOP (cyclophosphamide, doxorubicin, vincristine and prednisolone); (2) surgical treatment: it's primarily aimed at obtaining pathological information and confirming the diagnosis. It involves either removing the affected adnexa or performing a total hysterectomy with bilateral adnexectomy. It does not advocate for expanding the scope of surgery, and chemotherapy is the main focus of the treatment process; (3) combined therapy: it refers to preoperative neoadjuvant chemotherapy or preoperative concurrent chemoradiotherapy. The study by Signorelli et al. (13) noted a higher rate of complete remission in patients who received chemotherapy alone (75%) than in those who received postoperative chemotherapy (42%), suggesting that chemotherapy alone is more effective compared to surgery combined with chemotherapy. And chemotherapy, which preserves a patient's fertility function, is now the primary treatment option. Rituximab, an anti-CD20 monoclonal antibody, in combination with CHOP improves overall survival in DLBCL (9). Our patient was treated with 6 cycles of R-CHOP and 2 cycles of rituximab with excellent results and no signs of relapse.

Prognosis of uterine DLBCL is related to age, stage according to the Ann Arbor system (5) and treatment modality (14), and is also closely related to the expression of immunohistochemical markers. The 5-year survival rates of CD10 positive and Bcl-6 positive are higher, at 88.9% and 81.8%, respectively. However, the 5-year survival rates of CD10 negative and Bcl-6 negative are lower, at 25.0 and 33.3%, respectively (12). El-Galaly et al. (15) reported that 41% of patients with uterine DLBCL had secondary central nervous system (SCNS) involvement after R-CHOP(-like) treatment, whereas patients with ovarian-only involvement did not have SCNS. This symptom normally occurs within 2 years of treatment (16). Therefore, in addition to systemic chemotherapy, the prevention of CNS relapse should be considered during the course of treatment.

## Conclusions

Uterine DLBCL is a rare disease with a low incidence and no specific clinical presentation, making it relatively difficult to diagnose clinically. The ultrasound and MR can show some characteristic changes, and pathological biopsies and inventories can clarify diagnosis and reduce unnecessary surgical treatments. The R-CHOP chemotherapy regimen is effective in the treatment of this disease and preserves reproductive function in patients, and has become a first-line treatment option. Early diagnosis and treatment are important for this disease.

## Data availability statement

The original contributions presented in the study are included in the article/supplementary material, further inquiries can be directed to the corresponding author.

## Ethics statement

The studies involving human participants were reviewed and approved by the Institutional Review Board of Shengjing Hospital of China Medical University (approval number: 2023PS893K). The patients/participants provided their written informed consent to participate in this study. Written informed consent was obtained from the individual(s) for the publication of any potentially identifiable images or data included in this article. Written informed consent was obtained from the participant/patient(s) for the publication of this case report.

## Author contributions

ZZ was responsible for manuscript writing, literature analysis, and data collection. QY provided the knowledge of the disease and was responsible for important revisions of the manuscript. Final manuscript read and approved by all authors.
